# Osteonecrosis in Systemic Lupus Erythematosus: An Early, Frequent, and Not Always Symptomatic Complication

**DOI:** 10.1155/2012/725249

**Published:** 2012-08-05

**Authors:** Paola Caramaschi, Domenico Biasi, Ilaria Dal Forno, Silvano Adami

**Affiliations:** Rheumatology Unit, Department of Medicine, University of Verona, 37134 Verona, Italy

## Abstract

Osteonecrosis may complicate the course of systemic lupus erythematosus and may contemporaneously affect multiple joints. The major risk factor associated with the development of osteonecrosis is the use of glucocorticoid at high doses. Recent studies using serial MRI, which represents the “gold standard” for the early detection of osteonecrosis, yielded some interesting findings about the natural history of this clinical entity. Osteonecrosis in the majority of the cases is asymptomatic and occurs early in the course of the disease. Its later occurrence is associated with lupus flare that requires the increase of corticosteroid dose. The optimal treatment of osteonecrosis is controversial. In case of silent osteonecrosis involving a small area conservative strategy is usually adequate. When lesions are symptomatic surgical treatment as core decompression or free vascularized fibular grafting is required; extracorporeal shockwave treatment may represent an alternative therapeutic approach. When the lesion has a medium-large dimension or involves a weight-bearing area bone collapse is a common complication requiring total joint replacement. Coadministration of bisphosphonate or warfarin with high doses of corticosteroid might be a promising preventive strategy of osteonecrosis.

## 1. Introduction

Osteonecrosis is a clinical entity characterized by death of bone marrow and trabecular bone as a result of a compromised artery supply. It may follow a trauma resulting in the displacement of a bone fragment that disrupts blood supply causing bone ischemia (posttraumatic osteonecrosis) or may complicate the course of many systemic diseases or conditions (atraumatic osteonecrosis). In a proportion of osteonecrosis a triggering event cannot be identified (idiopathic forms).

Osteonecrosis mainly affects long-bone epiphyses, in particular femoral head and condyles, distal end of the tibia, and humeral head and often shows a multifocal distribution. Systemic conditions known as risk factors for osteonecrosis are corticosteroid treatment, Cushing syndrome, organ transplant, systemic lupus erythematosus (SLE), antiphospholipid antibody syndrome, thrombophilia, alcoholism, dyslipidemia, Caisson disease, sickle cell disease, Gaucher disease, HIV infection, pancreatitis, inflammatory bowel diseases, and pregnancy [1, 2].

Osteonecrosis may be silent or may clinically present with a gradual onset characterized by mild or vague pain that may worsen after ambulation and subsequently progress to severe pain when bone collapse occurs; in other cases osteonecrosis may cause a sudden onset deep joint pain that initially is elicited by movement and later is present also at rest.

Osteonecrosis may be asymptomatic and not progressive when a small fragment of bone tissue is involved. In case of medium-large bone area involvement usually the lesion progresses and eventually the necrotic tissue collapses. The clinical implication is a severe joint damage requiring arthroplasty. Negative prognostic factor is also the involvement of a weight-bearing area, such as the femoral head.

During the recent years magnetic resonance imaging (MRI) has become the “gold standard” technique for the early recognition of osteonecrosis considering the high sensitivity and specificity; it may detect early stage, silent osteonecrotic lesions, which are not diagnosed by both plain radiography and bone scan. Osteonecrosis is defined by MRI image of low-intensity band (band-like pattern) on T1-weighted images [3] which is attributed to the reaction at the interface between dead and normal bone.

In SLE the prevalence of symptomatic lesions in studies carried when MRI was not available varied from 2.1 to 30% [4–16]. More recent MRI studies allowed the diagnosis of osteonecrotic lesions in higher percentage of the patients. By serial MRI evaluation of hips and knees in 72 SLE patients on high doses of corticosteroid therapy osteonecrosis was observed in 32 subjects (44%), with a multifocal distribution in the majority of the cases. Thus 92 joints had osteonecrosis features, with a slightly predominance of knee involvement [17]. In all cases the bone lesions were asymptomatic at the time of the diagnosis and during the one year followup.

Osteonecrosis often involves more than one skeletal site; a case of SLE with twelve simultaneous osteonecrotic lesions was reported [18]; long-bone epiphyses are usually involved, but flat bones as ilium, sternum, talus, and vertebral bodies may be affected too [10, 19]. When osteonecrosis at one skeletal site is found in a SLE patient, a screening for other concomitant lesions is generally recommended since multifocal involvement is not infrequent.

Symptomatic osteonecrosis does not increase mortality but heavily impacts on quality of life and causes a significant physical disability as measured by the Health Assessment Questionnaire [16].

Progression from clinically occult osteonecrosis detected by MRI to symptomatic lesions is not frequent [10, 20]. A review about the natural history of asymptomatic osteonecrosis of femoral head showed that patients affected by SLE presented the most benign course, while in subjects suffering from sickle cell anemia the rate of progression to symptomatic disease and/or femoral head collapse was the highest [21].

Among a cohort of 500 SLE cases 19 patients had undergone at least one total joint replacement. In most of these cases rheumatoid arthritis was associated to SLE [22].

## 2. Skeletal Manifestations in Systemic Lupus Erythematosus

SLE is a complex autoimmune disease that may involve any organ and apparatus; it is characterized by great heterogeneity and the clinical picture may vary considerably from patient to patient and in the same subject from the onset to the following course. The involvement of the bone tissue includes osteoporosis and osteonecrosis, both unrelated to the autoimmune pathophysiology of the disease. The etiology of osteoporosis is multifactorial: corticosteroid treatment, limited physical activity and premature ovarian failure favoured also by cyclophosphamide treatment courses may contribute to bone loss [23]; chronic systemic inflammation per se may favour bone loss through increased production of TNF, a cytokine that influences osteoclast maturation and activity [24]. High levels of oxidized LDLs promote the activation of both T cells and the transcription factor peroxisome proliferator-activated receptor (PPAR)*γ*2 [25], a key regulator of both osteoblast and adipocyte differentiation from mesenchymal stem cells, which reduces the osteoblast precursor number in favour of the adipocyte cell number [26]. SLE patients are invariably encouraged to avoid sunshine exposure and this increases the risk of vitamin D deficiency.

## 3. Pathogenetic Mechanisms That Lead to Atraumatic Osteonecrosis

Although the pathogenesis of nontraumatic osteonecrosis is not completely understood, it is known that many factors may induce bone ischemia and favour bone necrosis by intraluminal or extraluminal obliteration [1]. Intraluminal occlusion may be due to the release of nitrogen gas bubbles that impinge the blood vessels as occurs in acute decompression syndrome or Caisson disease or may be provoked by fat embolism, which is favoured by dyslipidemia and glucocorticoid treatment or by thrombosis. A thrombophilic status has been associated with osteonecrosis. In a study that evaluated 45 cases of osteonecrosis 82.2% and 46.7% of the patients showed at least one and two risk factors for thrombosis, respectively, in comparison with 30% and 2.5% of the controls [27]. It has also been reported that patients affected by osteonecrosis are more likely to have hypofibrinolytic 4G polymorphism of the plasminogen activator inhibitor-1 gene, methylenetetrahydrofolate reductase gene mutation with higher concentration of homocysteine, low protein S values, and high lipoprotein(a) levels than controls [28]. Sickle vasoocclusion in sickle cell anaemia is another mechanism which may lead to intraluminal obliteration causing both symptomatic and asymptomatic osteonecrosis, which frequently complicates the course of the disease, requiring joint replacement in many cases [29].

Extraluminal obliteration is due to bone marrow pressure elevation, which impairs intraosseous blood flow because of the bone inelasticity. This mechanism, first hypothesized in 1972 [30], may be due to glucocorticoid treatment and alcohol abuse that cause bone marrow adipocyte hypertrophy.

Chronic corticosteroid administration promotes fat conversion of red marrow, as demonstrated by a MRI study of the proximal femur in a cohort of premenopausal women affected by SLE. The magnitude of fat conversion paralleled the daily prednisolone intake and was more evident in patients who had had osteonecrosis. The increase of bone marrow fat content may elevate marrow pressure reducing arterial perfusion [31]. Other mechanisms leading to increase of marrow pressure are bone edema, bleeding or histiocyte proliferation, typical histologic feature of Gaucher disease. All these observations suggest that the so-called atraumatic osteonecrosis represents the final common pathway of many different conditions leading to impaired bone blood supply.

A nationwide epidemiologic survey performed in Japan in 2005 permitted to obtain clinical data from 1502 patients with atraumatic osteonecrosis of the femoral head. Systemic glucocorticoid therapy and alcoholism were the most frequent risk factors, being present in 51% and 31% of the cases, respectively. SLE represents the most frequent underlying disease in steroid-induced osteonecrosis (31.2% of the cases), followed by nephrotic syndrome (6.3% of the cases); polymyositis/dermatomyositis, thrombocytopenic purpura, bronchial asthma, and inflammatory eye diseases were the underlying illness in about 4% of the patients, whereas rheumatoid arthritis in less than 1%. These proportions clearly do not mirror the epidemiology of the diseases. The most common disorders requiring corticosteroid treatment are bronchial asthma and rheumatoid arthritis, where osteonecrosis is relatively rare. The high incidence of osteonecrosis in SLE, nephrotic syndrome, and other autoimmune diseases might be related to the higher corticosteroid doses used but also to the underlying disease [32].

In a large Japanese survey it was found that patients who receive glucocorticoid are at approximately 20-fold greater risk to develop osteonecrosis in comparison with nonusers [33]. Glucocorticoid administration was also found to be a significant risk factor for the rare atraumatic osteonecrosis of the distal radius and ulna [34].

In a recent large study on the incidence of osteonecrosis among patients requiring corticosteroid treatment it was reported that daily prednisone-equivalent dose > 40 mg/day in comparison with lower doses was associated with an OR of 4.2 to develop osteonecrosis [35]. It was also found that male sex was an additional risk factor for the development of the disease.

Besides the aforementioned effects of corticosteroids on fat content of bone marrow, these drugs may impair bone integrity by inducing apoptosis of osteoblasts and osteocytes [36] and through the downregulation of osteoprotegerin and the upregulation of RANKL impairing the balance between bone formation and bone resorption [37]. Even tough a genetic predisposition has never been reported, the association with some polymorphisms has been found. The multidrug resistance 3435 TT haplotype was found to be protective for the development of osteonecrosis both in SLE patients [38] and in renal transplant recipients [39]. Interestingly this haplotype is associated with a reduced intracellular concentration of corticosteroids.

## 4. Factors Associated with the Development of Osteonecrosis in Systemic Lupus Erythematosus

The use of corticosteroid therapy is the major risk factor for the development of osteonecrosis in SLE [4, 17, 40–42].

The yearly incidence of osteonecrosis at hips and knees was prospectively investigated by MRI in a large cohort of patients requiring corticosteroid treatment for a variety of underlying diseases. MRI osteonecrosis (most often asymptomatic) developed in 255 (37%) of 687 joints evaluated in 173 SLE cases as compared with 107 (21%) of 512 joints in 129 non-SLE cases. The highest dose of corticosteroid was slightly different between SLE patients and subjects affected by other diseases (56.0 and 51.3 mg/day, resp.). The logistic regression analysis revealed that SLE patients had a higher risk to develop osteonecrosis versus other autoimmune or systemic diseases with an OR of 2.6. These results further confirm that SLE per se is a risk factor for osteonecrosis [35].

Vascular involvement, altered lipid metabolism, haemostatic abnormalities, and thrombophilia due to antiphospholipid antibodies have been considered as additional risk factors for the development of osteonecrosis in SLE. These conditions are generally reflecting a very high disease activity requiring high glucocorticoid dosages. However, contrasting data have emerged from studies focused on the association between osteonecrosis and disease activity. Fialho et al. reported a positive correlation between the development of osteonecrosis and the SLEDAI score (an integrated index of disease activity) ≥8 in the year preceding the clinical diagnosis of osteonecrosis [43]. On the contrary other studies did not find a correlation with disease activity [4, 5, 40] or severity [41].

Inconclusive results were yielded by analyzing the association of osteonecrosis with some typical features of SLE like vasculitis [4, 8], Raynaud's phenomenon [4, 6, 9], antiphospholipid antibody positivity [5, 6, 8, 10, 40, 44], and with administration of antimalarial drugs [4, 11]. Many authors reported a higher frequency of Cushingoid body habitus among SLE patients who developed osteonecrosis [8, 45, 46].

Osteonecrosis is relatively uncommon in growing individuals but its incidence rapidly increases in adolescents up to the rate seen in adults [35]. This may be related to the growing proportion of fatty marrow with advancing age. Fatty marrow as compared with red marrow has less collateral flow and is then more vulnerable to ischemic injury whereas children appear to better tolerate ischemia because they have abundant vascularity due to the sinusoidal network in hematopoietic red marrow [47]. A higher blood supply of femur head in pediatric patients after corticosteroid administration was demonstrated by dynamic MRI evaluation [48].

Another study on the prevalence of MRI detected osteonecrosis was carried out in Japan by Nakamura et al. Osteonecrosis MRI features were found in 260 of 676 joints (38%) in 169 SLE patients on treatment with corticosteroids. The authors ranked their cohort according to age: pediatric (<15 years old), adolescent (15–20 years old), and adult (>20 years old) with a prevalence of necrosis of 6%, 49% and 41% of the joints, respectively. None of the patients younger than 14 years developed osteonecrosis. These results indicate that patient age at the time of corticosteroid therapy initiation is an independent risk factor for osteonecrosis in SLE [49]. In a similar study in recipients of kidney transplantation no osteonecrosis cases were observed in children younger than 10 years [50].

The presence of arthritis [4], renal involvement [11], and the prevalence of anti-DNA and of various anti-ENA antibodies [5] could not be identified as risk factors for osteonecrosis. No differences in lipid profile were observed between SLE patients with and without osteonecrosis [11, 43, 44]. In a number of studies the incidence of osteonecrosis was significantly associated with the extensive use of immune suppressants drugs [4, 5, 44].

The aforementioned studies on the prevalence/incidence of osteonecrosis in SLE cannot be easily compared for their differences regarding important factors, such as age, duration of disease, duration of followup, dose, type, and route of administration of glucocorticoids. The definition of osteonecrosis was also different. It ranged from “clinical osteonecrosis” (the diagnosis having been achieved for the appearance of symptoms), X-ray, and eventually MRI-detected disease. For the latter it is not yet clear to what proportion these patients will develop full clinical picture of the disease, particularly keeping into account that effective treatment able to prevent the worsening of the disease is still lacking. What remains well established in all studies is the crucial role played by corticosteroid therapy in SLE patients.

## 5. Relationship between Osteonecrosis Development and Type of Corticosteroid Treatment

The relationship between development of osteonecrosis and corticosteroid treatment has been extensively investigated. Important risk factors appear to be the cumulative dose even for treatment cycles [5, 7, 9], the peak daily dose [5, 8, 44], the mean daily dose [5, 46], and the duration of use of high doses [51]. The use of methylprednisolone pulses was reported to be associated with osteonecrosis [38, 45], but this has not been confirmed by others [5, 16].

In all aforementioned studies the diagnosis of osteonecrosis was clinical and supported by traditional X-ray.

The epidemiological scenario has completely changed when the presence of osteonecrosis was identified also in asymptomatic patients by MRI. The use of this technique changed substantially the epidemiology of osteonecrosis and provided also interesting and unexpected data regarding the natural history of osteonecrosis in SLE. For example, in one of these studies it was found that osteonecrosis may occur soon after the beginning of corticosteroid therapy; all lesions were present within five months (on average after 3.1 months), and no new cases were observed afterward! In this particular study the corticosteroid dose was not significantly associated with osteonecrosis [17].

These results are somewhat supported by another interesting study prospectively evaluating by repeated MRI 291 joints in 106 SLE patients without osteonecrosis after the initial corticosteroid treatment. During the long follow-up period (on average 13.6 years) incidental osteonecrosis was observed only in 6 bone segments. In all these cases the osteonecrosis developed after a SLE flare requiring the increase of corticosteroid dosage. No cases of osteonecrotic lesions were found in patients who received a low or medium corticosteroid dose, equivalent to <30 mg/day of prednisone. Thus, it appears that if a SLE patient does not develop osteonecrosis after an initial course of high steroid therapy he will rarely present the complication afterwards unless the dose of corticosteroid is considerably increased as a consequence of a disease flare [52].

A study was focused on the MRI evolution over 10 years in SLE patients who already suffered from a noncollapsed and asymptomatic osteonecrosis at the hips or knees (238 (44%) out of 537 joints). During the followup in about half of the lesions spontaneous reduction of necrotic areas was observed with complete regression in 9% of the cases. Progression of the osteonecrotic lesions occurred in 14% of the cases, invariably associated with an increase of corticosteroid dosage due to SLE recurrence [53].

## 6. Treatment

The treatment of osteonecrosis in SLE patients is similar to that of osteonecrosis due to other causes. The goal of the treatment of osteonecrotic lesions is to preserve joint integrity by preventing bone collapse.

When osteonecrosis involves less than 10% of the femoral head or less than 1/3 of the weight-bearing portion the outcome is usually favourable and surgical treatment is not required [1]; the conservative treatment includes analgesics and the use of devices to allow nonweight bearing; these measures are usually effective in alleviating pain.

In case of symptomatic lesions surgical treatment is usually required.

Core decompression technique was performed for the first time in 1962 in order to reduce bone marrow pressure and to improve perfusion of ischemic bone [54]. Mont et al. treated by core decompression 31 hips in 18 SLE patients; in 21 cases (68%) a subsequent total joint replacement was required; all these cases were in an advance stage of the disease. The authors concluded that an early detection of the lesion and a prompt treatment are crucial to obtain optimal results [55]. Core decompression is usually ineffective when osteonecrosis involves more than 25% of the femoral head or more than 2/3 of the weight-bearing portion [1]. Core decompression was successfully performed in cases of osteonecrosis of the knee and of the talus in the precollapsed stages [55, 56].

Concentrated autologous bone marrow aspirate transplantation following core decompression was successfully performed in 8 out 9 SLE cases with osteonecrosis of the femur head involving more than 2/3 of the weight-bearing portion; only the patient who showed an advanced stage lesion failed to obtain benefit and later required joint replacement. Non perioperative complications were observed [57].

Another option is free vascularised fibular grafting; this procedure was applied in 80 osteonecrotic lesions of the hip among 50 SLE patients who were followed at least for two years after the procedure, on average for 4.3 years; none of the cases required hip arthroplasty, suggesting that free vascularised fibular grafting may allow to maintain joint function [58].

Transtrochanteric anterior rotational osteotomy demonstrated to be effective in a long-term study, showing a hip survival rate of 73.7% after 25 years from the surgical approach [59].

A 19-year-old woman affected by SLE who developed bilateral femur osteonecrosis that affected more than 2/3 of the weight-bearing area was treated with extracorporeal shockwave under general anesthesia obtaining significant pain relief, hip functional amelioration, and reduction of bone edema [60]. Shockwave treatment may favour neovascularization through an increased release of angiogenic factors [61] and may enhance bone mass and bone strength as demonstrated in rabbits [62]. After this first favourable experience, 15 SLE patients with 26 hips affected by osteonecrosis were treated with extracorporeal shockwave; hip replacement was necessary in 12% of the cases. The outcome was similar to that observed in a group of non-SLE patients with osteonecrosis [63].

The outcome of hip arthroplasty in SLE-associated osteonecrosis is not different from that observed in other indications; an overall survival probability of 94.6% at 5 years and of 81.8% at 9 years was reported with minimal perioperative morbidity [64]. In 19 SLE patients who underwent 26 hip arthroplasty interventions, two early, nonrecurrent dislocations, and one low-grade prosthetic infection were described [65].

Less favourable results were obtained for knee arthroplasty in SLE patients with osteonecrosis; a good outcome was reported in 11 out of 25 cases. No differences were found when patients were stratified by amount of corticosteroid use, cemented versus cementless fixation, and SLE activity [66].

Alendronate 70 mg per week was tested in a randomized placebo controlled study in 40 patients affected by unilateral or bilateral nontraumatic osteonecrosis of the femoral head with a necrotic area larger than 30%. Only 2 out of 29 femoral heads collapsed in the group treated with alendronate in comparison with 19 out of 25 femoral heads in the control group. Joint replacement was necessary in 1 alendronate-treated patient as compared with 16 cases of the placebo group [67]. A prospective study evaluating the effectiveness of the contemporaneous administration in SLE patients of high corticosteroid dose with bisphosphonate should be planned in order to establish if this class of drugs may prevent the onset or may halt the progression of osteonecrotic lesions.

The preventive strategy for osteonecrosis development in SLE patients requiring high corticosteroid dose to control disease activity has been seldom studied. Sixty newly diagnosed SLE patients treated with ≥40 mg of prednisolone daily were alternatively assigned to two options, a warfarin group and a control group; warfarin was given together with the initiation of corticosteroid therapy. Silent and symptomatic osteonecrosis developed in 21% and 4.8% of the hips in the warfarin group, respectively, and in 33% and 14% of the hips in the control group, respectively. Despite the lack of a statistically significant difference, the observed results indicate that larger studies are warranted in order to verify the efficacy of anticoagulation therapy for preventing osteonecrosis at least in SLE patients [68].

According to the actual understanding of the pathophysiology of osteonecrosis, some preventing benefits might be expected from the treatment with lipid lowering agents, antiplatelet drugs, and antiresorptives. However, in a small study in SLE patients with disease flare requiring increased corticosteroid dosage, no beneficial effects were observed with the concomitant administration of statin, antiplatelet, and bisphosphonate [69].

## 7. Conclusion

Osteonecrosis frequently complicates the course of SLE, usually occurs soon after the initiation of corticosteroid therapy at high doses or, more rarely, after increasing the dosage for SLE flares. The complication may have a significant impact on functional ability and may require in some cases total joint replacement.

The presence of localized asymptomatic forms can be nowadays identified by MRI. By using this technique the incidence of osteonecrosis in SLE patients appears to be considerably higher than hitherto thought.

Recent MRI serial evaluation of hips and knees shed more light on the natural history of osteonecrosis, which does not progress if the dose of corticosteroid is maintained low and possibly thanks to promising preventive strategies, such as warfarin and bisphosphonates. However, at the moment the best documented preventive strategy is a judicious glucocorticoid use which is now possible by adopting steroid-sparing therapies, including the new biological agents.
